# Supplementary material for: Context-specific effects of sequence elements on subcellular localization of linear and circular RNAs
Source: Nat Commun. 2022 May 5;13:2481. doi: 10.1038/s41467-022-30183-0 (PMC9072321; doi:10.1038/s41467-022-30183-0)
Supplement: Supplementary file 1 — Supplementary Information [file 41467_2022_30183_MOESM1_ESM.pdf]

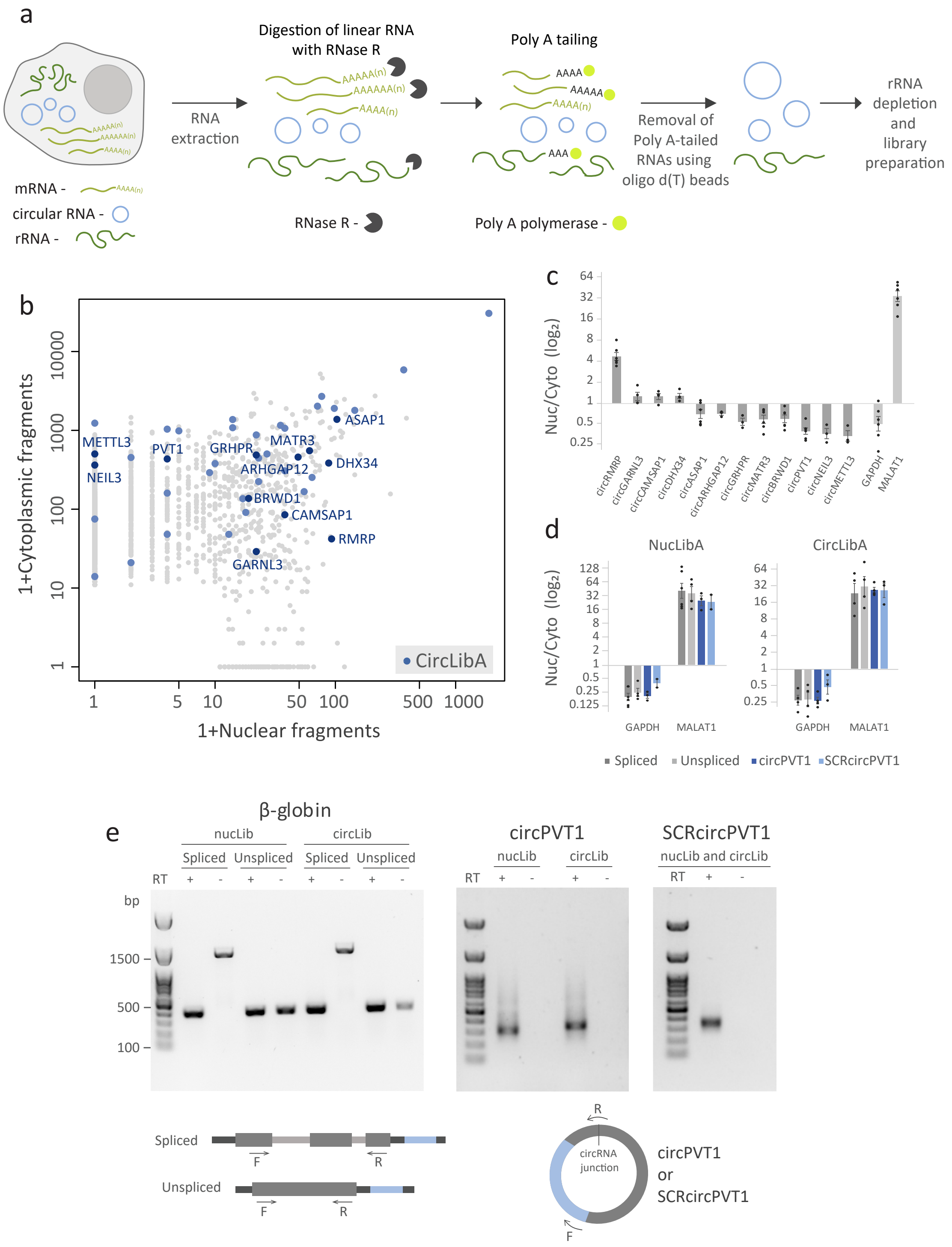

a
Digestion of linear RNA with RNase R
Poly A tailing
RNA extraction
Removal of Poly A-tailed RNAs using oligo d(T) beads
rRNA depletion and library preparation
AAAAA(n)
AAAAAA(n)
AAAA(n)
AAAA
AAAAA
AAA
mRNA -
circular RNA -
rRNA -
RNase R -
Poly A polymerase -
b
1+Cytoplasmic fragments
1+Nuclear fragments
1
5
10
50
100
500
1000
10000
METTL3
NEIL3
PVT1
GRHPR
MATR3
ARHGAP12
ASAP1
DHX34
BRWD1
CAMSAP1
RMRP
GARNL3
CircLibA
c
Nuc/Cyto (log2)
64
32
16
8
4
2
0.5
0.25
circRMRP
circGARNL3
circCAMSAP1
circDHX34
circASAP1
circARHGAP12
circGRHPR
circMATR3
circBRWD1
circPVT1
circNEIL3
circMETTL3
GAPDH
MALAT1
d
NucLibA
CircLibA
128
0.125
GAPDH
MALAT1
Spliced
Unspliced
circPVT1
SCRcircPVT1
e
β-globin
nucLib
circLib
Spliced
Unspliced
RT
bp
1500 –
500 –
100 –
circPVT1
SCRcircPVT1
nucLib and circLib
F
R
circRNA junction
circPVT1 or SCRcircPVT1

Supplementary Figure 1 - CircLibA design and baseline localization of reporters. a Outline of the RPAD protocol; RNA samples are treated with RNase R, followed by treatment with poly A polymerase. polyA-tailed RNAs are eliminated using oligo-d(T) coated beads. Libraries for sequencing are generated using a library preparation kit based on rRNA depletion. **b** Number of fragments supporting the expression of circular RNAs in the nuclear and cytoplasmic fractions. Each point is a circRNA annotated in one of the examined datasets (see Methods). circRNAs selected for tiling in CircLibA are in blue. **c** qPCR analysis of nuclear and cytoplasmic fractions of selected circRNAs. n=6 biologically independent samples. Selected circRNAs are marked in Supplementary Fig. 1B (dark blue). Data are presented as mean values +/- SEM. **d** qPCR analysis of nuclear and cytoplasmic fractions following transfection of NucLibA (left, n=3) and CircLibA (right, n=4). Data are presented as mean values +/- SEM. **e** Splicing efficiency of the reporter mRNAs. Unspliced and spliced vectors: β-globin gene was amplified using primers that amplify a fragment that contains exons 1-3. Expected product size is 400 bp without introns and 1500 bp with introns. circPVT1 and SCRcircPVT1 vectors: circRNAs were amplified using a primer that spans the junction of the intended circRNA and a primer for the 5' adapter of NucLibA and CircLibA. Expected product size is 310 bp (for NucLibA) and 340 (for CircLibA). PCR was conducted using reverse transcriptase products (+RT) or RNA samples as control (-RT). Size of DNA ladder standards is labeled on the left. This experiment was repeated independently with similar results 3 times.

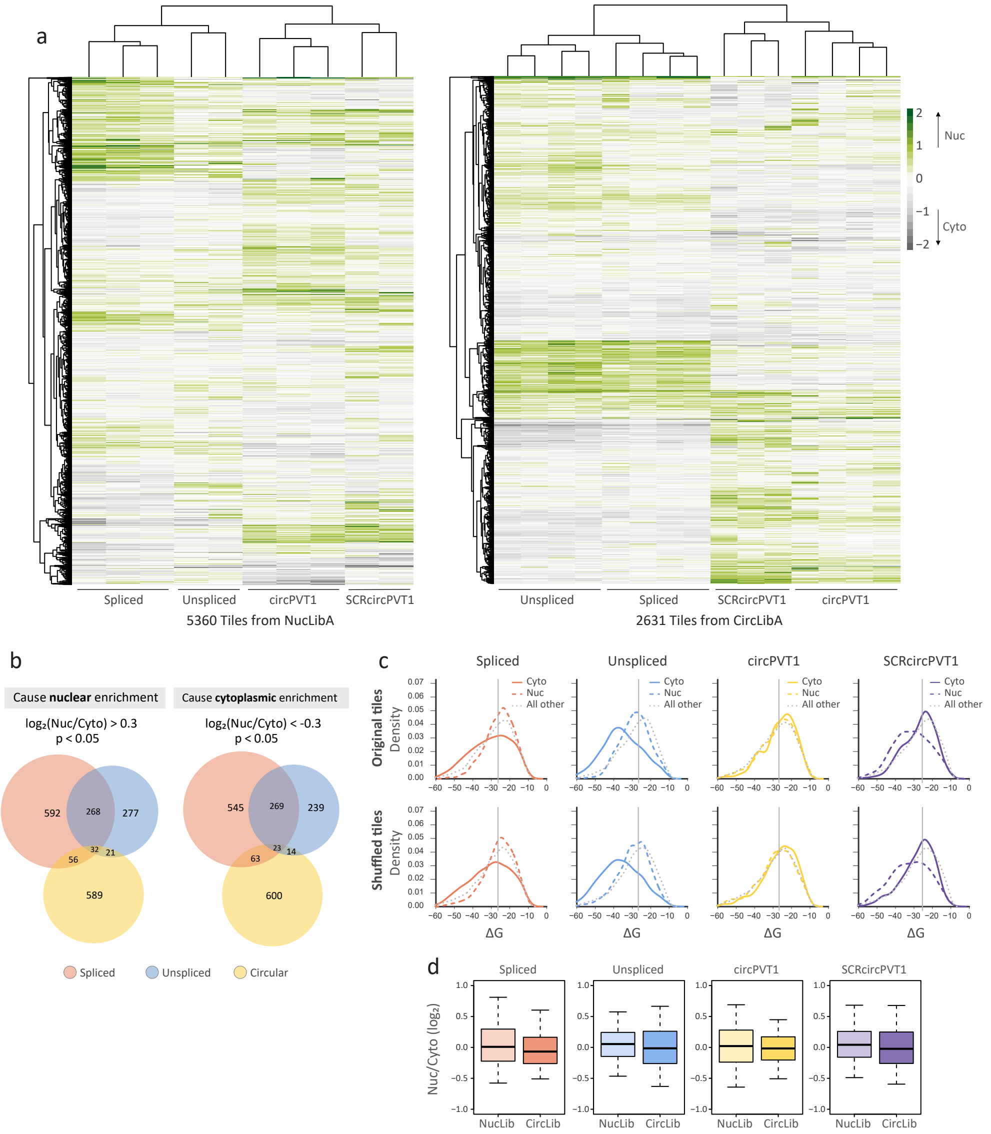

Supplementary Figure 2 – Technical characterization of the MPRNA results. a Subcellular localization of all tiles in all samples in the spliced, unspliced, circPVT1 and SCRcircPVT1 contexts. Color indicates the log_2(Nuc/Cyto) of each tile. Cytoplasmic shift is in grey, nuclear shift is in green. **b** Numbers of tiles that were significantly enriched in the cytoplasmic or in the nuclear fractions of each library and their overlap. P values computed using two sided Wilcoxon rank-sum test between the normalized counts in the cytoplasmic and nuclear fractions. **c** ΔG distributions of the tiles and of a dinucleotide-preserving shuffled version of all tiles, enriched in the cytoplasmic fraction (solid line), nuclear fraction (dashed line) or all other tiles (dotted line) in each context. Vertical gray line indicates the median of all tiles in the sample. **d** Nuc/Cyto ratios of NucLibA and CircLibA tiles in each context; spliced (red), unspliced (blue) and circular (yellow and purple). n=3 biologically independent samples. Box plots show median, first to third quartile, whiskers are 1.5× interquartile range.

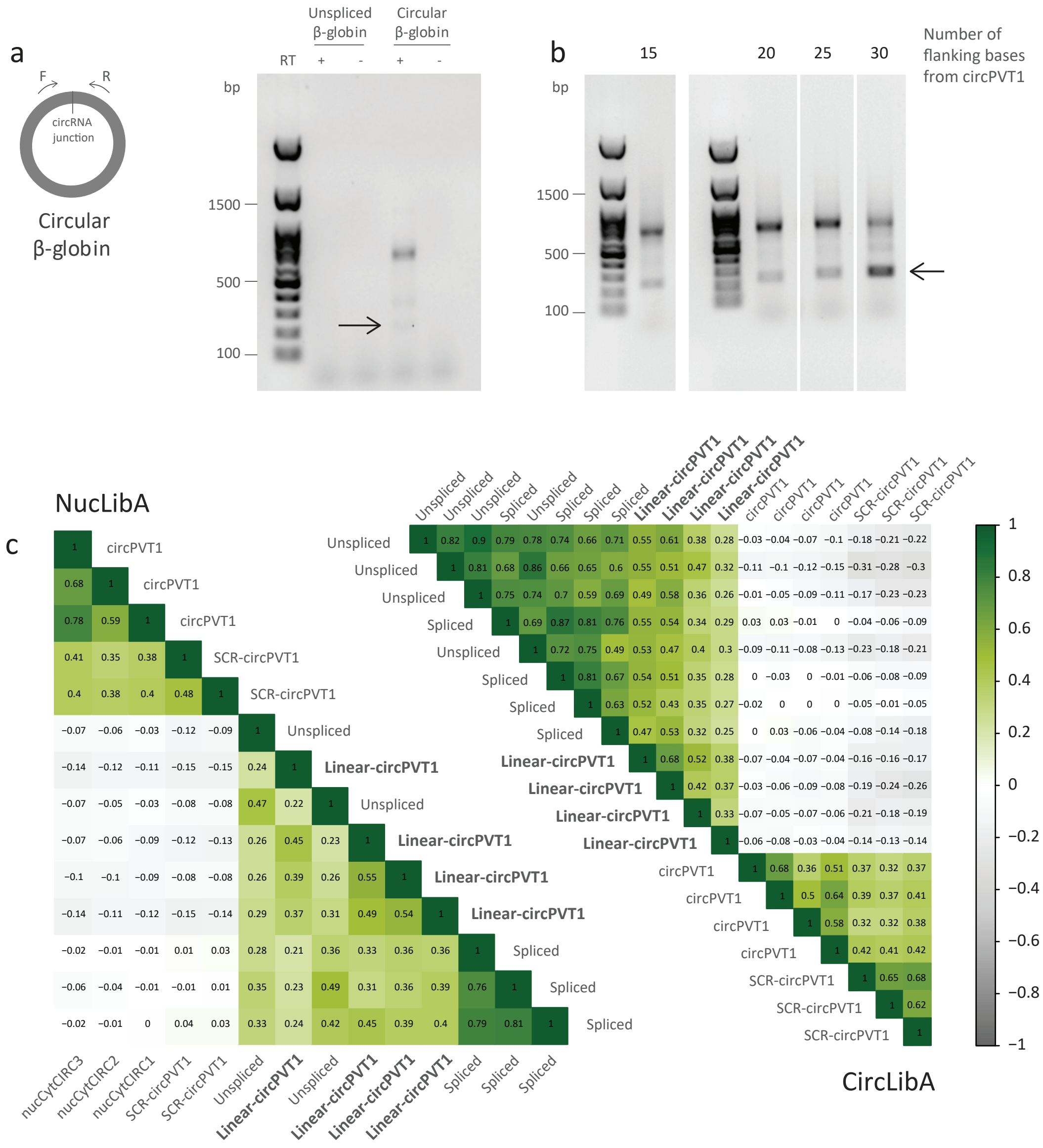

Supplementary Figure 3 - A circular vector encoding β-globin and linear-circPVT1. **a** Splicing efficiency of the reporter mRNA. Circ β-globin was amplified using primers that span the junction of the circRNA. The expected product length is 230bp, indicated by an arrow. This experiment was repeated independently with similar results 2 times. **b** Splicing efficiency of the reporter mRNA, after addition of the indicated number of flanking bases from circPVT1. The expected product length is 260-290bp, indicated by an arrow. This experiment was repeated independently with similar results 2 times. **c** Correlation plots for the localization of all tiles in each one of the contexts; Spliced, Unspliced, circPVT1, SCR-circPVT1 and Linear-circPVT1. Color-coded values indicate the pairwise Spearman's correlations between Nuc/Cyto ratios in each sample.

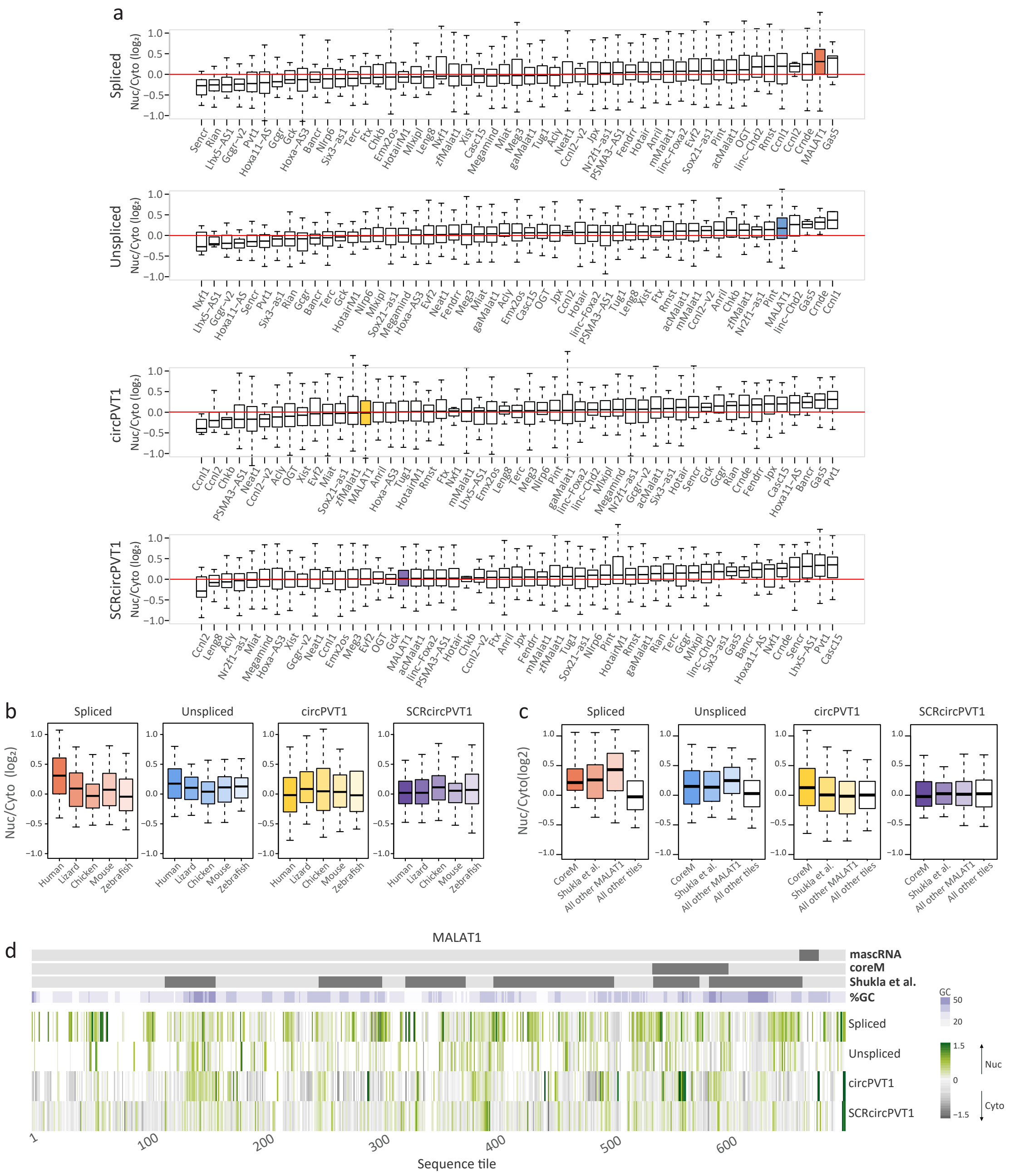

Supplementary Figure 4 – Nuc/Cyto ratios for groups of tiles derived from specific genes. a The localization of NucLibA tiles originating from individual genes as a group in each sample. The group of the human *MALAT1* gene is marked. n=3 biologically independent samples. Box plots show median, first to third quartile, whiskers are 1.5× interquartile range. **b** Nuc/Cyto ratios of tiles originating from human, lizard, chicken, mouse and zebrafish *MALAT1* gene in each context. n=3 biologically independent samples. Boxplots are as in a. **c** Nuc/Cyto ratios of tiles originating from specific regions within *MALAT1*. n=3 biologically independent samples. Boxplots are as in a. **d** Subcellular localization of the tiles derived from the lncRNA *MALAT1* in the spliced, unspliced, circPVT1 and SCRcircPVT1 contexts. Color indicates the $\log_2$(Nuc/Cyto) of each tile. Cytoplasmic shift is in gray, nuclear shift in green.

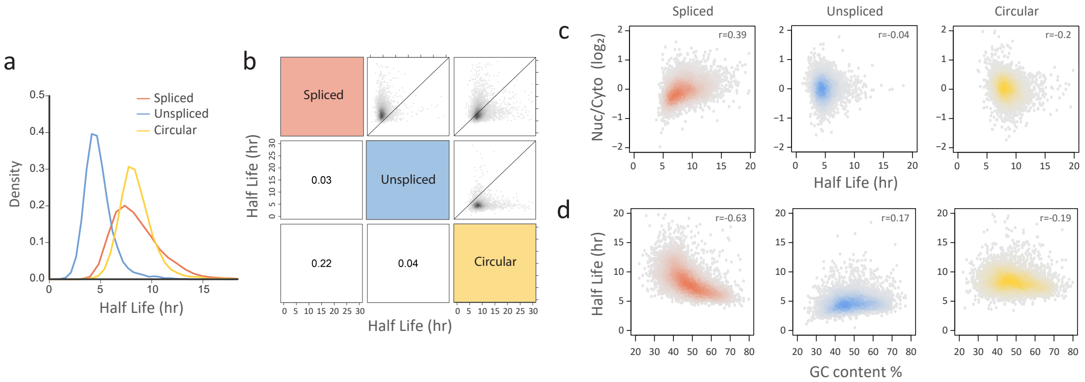

Supplementary Figure 5 – MPRNA for RNA stability. a Half-life distributions of tiles in each context. **b** Correlations between the half life of tiles in the spliced, unspliced and circular contexts. **c** The correlation between Nuc/Cyto ratio and half life of tiles in each context. **d** The correlation between G/C content and half life of tiles in each context.

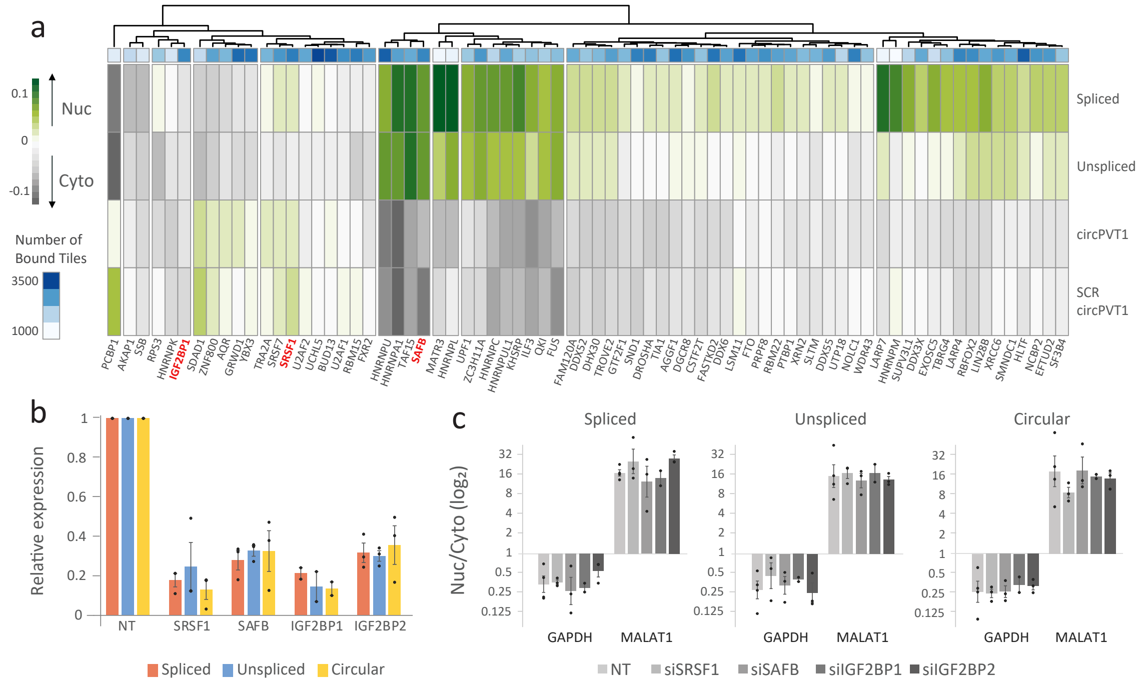

Supplementary Figure 6 – Identifying RBPs underlying tiles contributing to RNA localization. a Subcellular localization eCLIP analysis, using data from K562 and HepG2 cells profiled by the ENCODE project. Color indicates the difference between the log2(Nuc/Cyto) of tiles that are bound by a specific RNA binding protein (RBP) and all other tiles. Cytoplasmic shift is colored in gray, nuclear shift is colored in green. Selected RBPs are marked in red. **b** qPCR analysis of *SRSF1*, *SAFB*, *IGF2BP1*, or *IGF2BP2* KDs using siRNA pools in MCF-7. Normalized to non-targeting (NT) control. n=3 biologically independent samples, n=2 for IGF2BP1. Data are presented as mean values +/- SEM. **c** qPCR analysis of nuclear and cytoplasmic fractions following transfection of NT, siSRSF1, siSAFB, siIGF2BP1, and siIGF2BP2. (n=3. n=2 for IGF2BP1). Data are presented as mean values +/- SEM

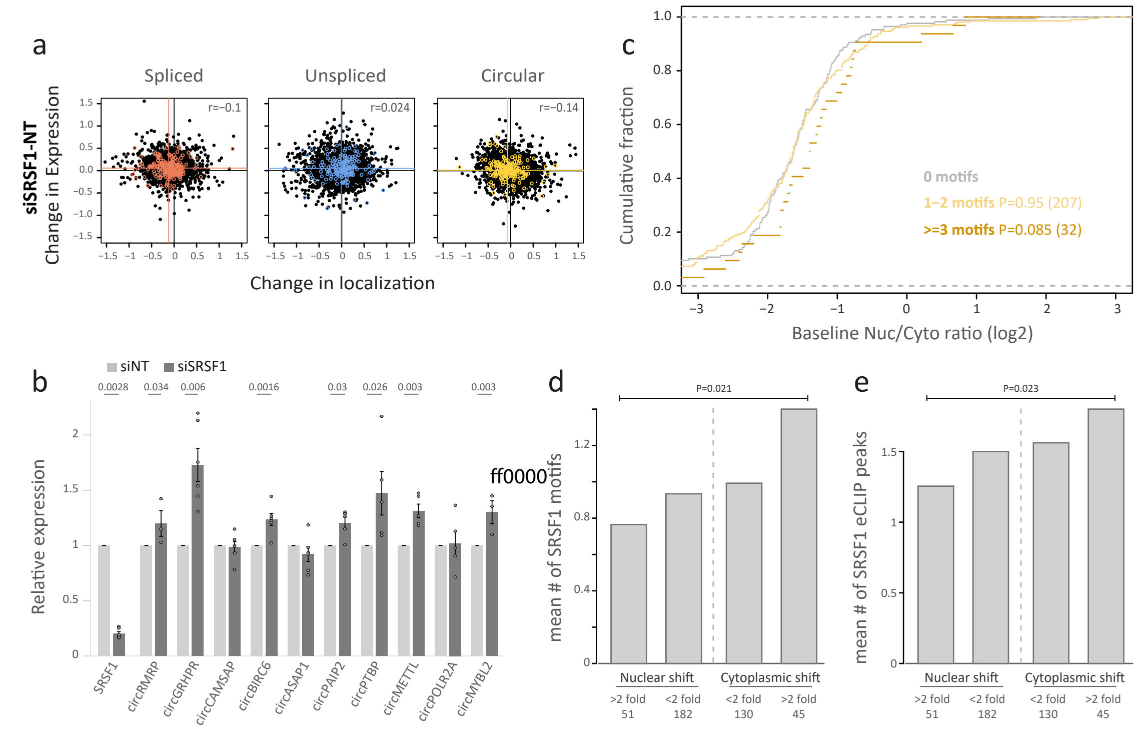

Supplementary Figure 7 - The effect of SRSF1 KD. a Correlation between the change in localization (X axis, normalized log_2(Nuc/Cytoa) values in the KD sample vs. control) and expression (Y axis, normalized WCE/input values in KDs samples vs. control) of all tiles (black) and tiles that have more than 1 SRSF1 eCLIP clusters (colored). Lines indicate X=0 and Y=0 (Black) and mean of all tiles that have more than 1 SRSF1 eCLIP clusters (colored). **b** qPCR analysis of circRNAs following KD of SRSF1. Normalized to NT control. n=6 biologically independent samples. Data are presented as mean values +/- SEM. **c** Baseline Nuc/Cyto ratios for circRNAs annotated in circBase with the indicated number of SRSF1 binding motifs. P-values computed using Wilcoxon rank-sum test, and the number of circRNAs in each group is indicated in parentheses. **d,e** Mean number of SRSF1 binding motifs **(d)** or eCLIP clusters **(e)** in the sequences of circRNAs with the indicated change in localization following KD of SRSF1. P-values computed using two sided t-test.

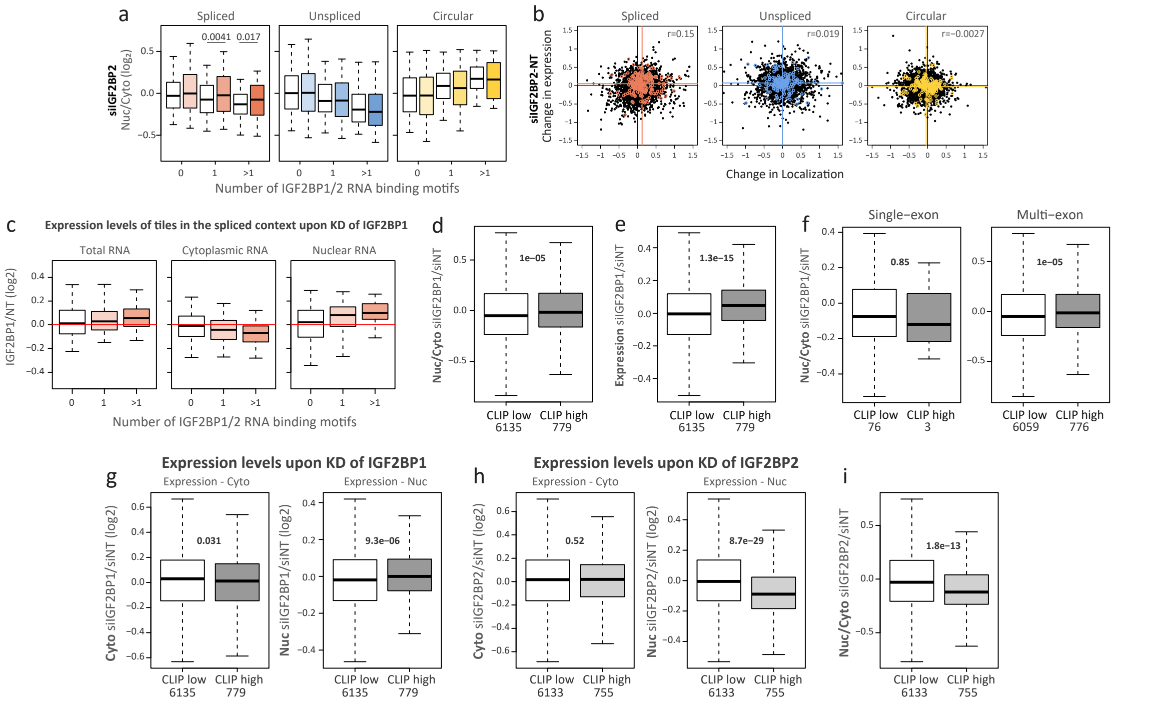

Supplementary Figure 8 - IGF2BP1 regulates nuclear export of linear spliced RNAs. **a** Nuc/Cyto ratio of tiles with the indicated number of IGF2BP1/2 motifs following transfection of NT control (white) and siIGF2BP2. n=3 biologically independent samples. Box plots show median, first to third quartile, whiskers are 1.5× interquartile range. **b** Correlation between the change in expression in the cytoplasmic fraction (X axis, normalized values in KDs samples vs. control) and in the nuclear fraction (Y axis, normalized values in KDs samples vs. control) of all tiles (black) and tiles that have more than one IGF2BP2 eCLIP cluster (colored). Lines indicate X=0 and Y=0 (Black) and mean of all tiles that have more than 1 IGF2BP2 eCLIP clusters (colored). **c** Change in expression of tiles with the indicated number of IGF2BP1 motif following KD of IGF2BP1 in the cytoplasmic and nuclear fractions. Boxplots are as in a. **d** Change in localization of transcripts with low (<3) or high (≥10) number of IGF2BP1 eCLIP clusters in K562 cells following KD of IGF2BP1 in MCF-7 cells. Number of genes in each group is specified below. Only genes expressed in both MCF-7 and K562 cells were considered. (n=2 for figures d-i). Boxplots are as in a. **e** Change in expression of genes with low or high number of IGF2BP1 eCLIP clusters (defined as in d) following KD of IGF2BP1. Number of genes in each group is specified. Boxplots are as in a. **f** Change in localization of single-exon transcripts (left) and multi-exon transcripts (right) with low or high number of IGF2BP1 eCLIP clusters following KD of IGF2BP1. Number of genes in each group is specified. Boxplots are as in a. **g-h** Change in expression of genes with low or high number of IGF2BP1 **(g)** or IGF2BP2 **(h)** eCLIP clusters following KD of IGF2BP1 **(g)** or IGF2BP2 **(h)** in the cytoplasmic and nuclear fractions. Number of genes in each group is specified below. Boxplots are as in a. **i** Change in localization of transcripts with low or high number IGF2BP2 eCLIP clusters following KD of IGF2BP2. Number of genes in each group is specified. Boxplots are as in a.

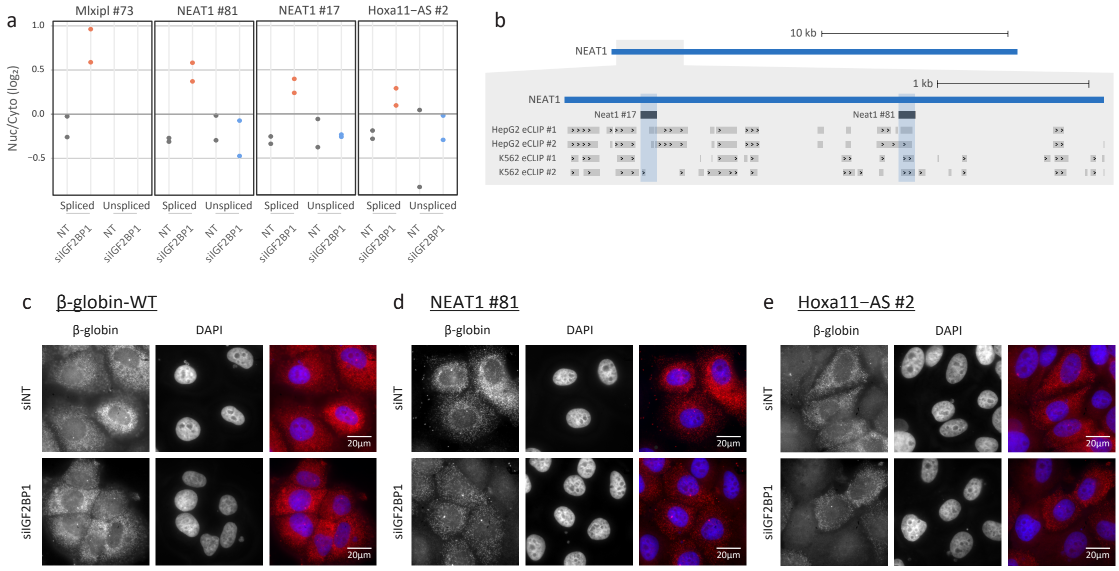

Supplementary Figure 9 - smFISH. a Nuc/Cyto ratios of Mlxipl#73, NEAT1#81, NEAT1#17 and HOX11AS#2 tiles in the spliced and unspliced contexts, in control and in IGF2BP1-depleted cells. **b** Genome browser view of the human NEAT1 locus, highlighting the positions of the NEAT1#17 and NEAT1#81 tiles, and clusters from the indicated eCLIP experiments by the ENCODE project. **c-e** Representative smFISH images of β-globin-WT (**c**), β-globin-Neat1 #81 (**d**) and β-globin-Hox11-AS #2 (**e**) and DAPI, in control and in IGF2BP1-depleted cells.
