# Supplementary material for: Context-specific effects of sequence elements on subcellular localization of linear and circular RNAs
Source: Nat Commun. 2022 May 5;13:2481. doi: 10.1038/s41467-022-30183-0 (PMC9072321; doi:10.1038/s41467-022-30183-0)
Supplement: Supplementary file 3 — Description of Additional Supplementary Files [file 41467_2022_30183_MOESM3_ESM.pdf]

Description of Additional Supplementary Files

File Name: Supplementary Data 1
Description: Design of CircLibA

File Name: Supplementary Data 2
Description: Oligos used for cloning and qPCR, siRNA sequences, and FISH probes

File Name: Supplementary Data 3
Description: - NucLibA and CircLibA analysis

File Name: Supplementary Data 4
Description: RNA-seq analysis

File Name: Supplementary Data 5
Description: Backbone sequences.
